# Carroll County Medical Society

**Published:** 1869-01-01

**Authors:** 


### EDITORIAL.

The somewhat extended absence of our Publisher from the city, and a similar, but temporary, journey of the Editor, must still be pleaded in extenuation of sins of omission on our part. We expect to be fully at rights soon, and meanwhile we chronicle, with pride, the fact that the monthly of old time has been transformed into a semi monthly, with abundant success. Only the editorial want of time has prevented its still further advance to hebdomadal issue—a thing which we still hope to accomplish within a brief period. A large number of valuable and interesting communications are on file for publication, for which our correspondents will accept thanks, and our patrons may look forward with pleasant anticipations.

To those of our subscribers who have cheered us by their friendly words and "material aid," we extend our sincerest thanks. To those few who leave us with the closing year, we send best wishes in their new avocations or associations. To the many who have sent us their names for the first time, we tender a hearty greeting, and promise to all renewed interest and a richer magazine of professional lore and experience, in the pages of the Journal for 1869.

Messrs Editors: Be kind enough to insert the following *errata* in your next Journal; they seem necessary to a correct knowledge of what was intended to be said in the recent article on Dystocia, written by

Your obedient servant, H. Webster Jones.

1. Last line of page 753, read for "votating," "rotating."
2. Fifth line from bottom of page 754, for "coincidentally" read "coincidently."
3. Fourth line from bottom of same page, for "secured" read "seemed," and omit comma.

### CARROLL COUNTY MEDICAL SOCIETY.

Pursuant to a call circulated among the physicians, belonging to the Regular Profession of Medicine and Surgery, in Carroll County, a meeting was held in the First Baptist Church, in Lanark, on Tuesday, December 8th, 1868, for the purpose of organizing a County Medical Society, for a more thorough protection, both in and out of the profession, against the evils of Medical Empiricism; and to afford mutual assistance to each other in the amelioration of the sufferings of humanity.

Dr. J. L. Hostetter was chosen temporary Chairman of the meeting. The Constitution, By-Laws, and Rules of Order were then read, and on motion were adopted, as the basis for the government of the Society. The officers for the coming year were then elected, as follows:

President, Dr. John L. Hostetter, of Mount Carroll; Vice-President, Dr. J. B. Porter, of Lenark; Secretary and Treasurer, Dr. J. Haller, of Lanark.

The following named members were, by resolution, requested to read papers at the next meeting of the Society, to wit:

On the action of Chloroform as an Anæsthetic Agent,—Dr. N. Stephenson, of Thompson.

On the action of Veratrum Viride as an Arterial and Nervous Sedative, and to what extent in dose may it be safely administered,—Dr. D. M. Greely, of Mount Carroll.

On Fractures in general, both compound and simple,—Dr. B. P. Miller, of Mount Carroll.

On the action of Gelseminum Sempervirens,—Dr. J. Haller, of Lanark.

On the History, Rise, and Progress of Medicine,—D. J. B. Porter, of Lanark.

On Medical Ethics in general,—Dr. J. L. Hostetter, of Mount Carroll.

On motion, it was resolved that any member of the Press, and Clergymen, who are favorable to the "Regular System of Medicine," be allowed seats at the meetings of this Society.

On motion, it was ordered that the Secretary make out and forward a report of this to the CHICAGO MEDICAL JOURNAL and Medical Examiner, for publication.

There was a good attendance of the physicians of the county, and a most cordial feeling prevailed. After an interchange of views on different topics of medicine, the Society adjourned to meet in Lanark, on Monday, February 22, 1869, at 2 o'clock, P. M. J. HALLER, *Secretary*.

THE Adams County Medical Society, at its November meeting, adopted a revised fee bill, together with the following:

In cases where consultations have been had, or where two or more physicians have been in attendance, the physician having the case in charge shall embody in his bill the fees of the counsel or assistants, and account therefor to the same, paying the full amount or such proportion of the whole bill as may be collected.

I. *Resolved*, That the forgoing table of fees be adopted as the Fee Bill of this Society.

II. *Resolved*, That the virtuous and industrious poor shall be attended by the members of this Society as cheerfully as the rich, and for such compensation as they are able to make.

III. *Resolved*, That every member of this Society shall keep a record of all patrons who are able, but who neglect or refuse to pay for medical services, and report the name and residence of the same at every meeting; and that all such persons be afterwards required, by all the members, to pay for services in advance.

IV. *Resolved*, That our fees are due as soon as the services are rendered, and that all members of the Society shall make prompt collections, and give the community to underetand that we are entitled to the same consideration in this respect that is claimed by merchants, artizans and laborers.

V. *Resolved*, That this Fee Bill, and these Resolutions, with the names of all the member of the Society appended thereto, be printed in a suitable form, and that every member be required to have a copy of the same conspicuously placed in his office, that our patrons may be fully apprised of its contents and conditions.

VI. *Resolved*, That members who disregard this Fee Bill, and these Resolutions, shall be liable to the same penalties that are attached to a violation of the Code of Ethics.

